# The Mechanism Underlying the Antibacterial Activity of Shikonin against Methicillin-Resistant *Staphylococcus aureus*

**DOI:** 10.1155/2015/520578

**Published:** 2015-07-21

**Authors:** Young-Seob Lee, Dae-Young Lee, Yeon Bok Kim, Sang-Won Lee, Seon-Woo Cha, Hong-Woo Park, Geum-Soog Kim, Dong-Yeul Kwon, Min-Ho Lee, Sin-Hee Han

**Affiliations:** ^1^Department of Herbal Crop Research, National Institute of Horticultural and Herbal Science, Rural Development Administration (RDA), Eumsung, Chungbuk 369-873, Republic of Korea; ^2^Department of Oriental Pharmacy, College of Pharmacy and Wonkwang-Oriental Medicines Research Institute, Wonkwang University, Iksan, Jeonbuk 570-749, Republic of Korea; ^3^Department of Food Technology and Services, Eulji University, Seongnam, Gyeonggi 461-713, Republic of Korea

## Abstract

Shikonin (SKN), a highly liposoluble naphthoquinone pigment isolated from the roots of *Lithospermum erythrorhizon*, is known to exert antibacterial, wound-healing, anti-inflammatory, antithrombotic, and antitumor effects. The aim of this study was to examine SKN antibacterial activity against methicillin-resistant *Staphylococcus aureus* (MRSA). The SKN was analyzed in combination with membrane-permeabilizing agents Tris and Triton X-100, ATPase inhibitors sodium azide and *N,N*′-dicyclohexylcarbodiimide, and *S. aureus*-derived peptidoglycan; the effects on MRSA viability were evaluated by the broth microdilution method, time-kill test, and transmission electron microscopy. Addition of membrane-permeabilizing agents or ATPase inhibitors together with a low dose of SKN potentiated SKN anti-MRSA activity, as evidenced by the reduction of MRSA cell density by 75% compared to that observed when SKN was used alone; in contrast, addition of peptidoglycan blocked the antibacterial activity of SKN. The results indicate that the anti-MRSA effect of SKN is associated with its affinity to peptidoglycan, the permeability of the cytoplasmic membrane, and the activity of ATP-binding cassette (ABC) transporters. This study revealed the potential of SKN as an effective natural antibiotic and of its possible use to substantially reduce the use of existing antibiotic may also be important for understanding the mechanism underlying the antibacterial activity of natural compounds.

## 1. Introduction

Among the emerging multidrug-resistant bacteria, methicillin-resistant* Staphylococcus aureus* (MRSA) is a major cause of nosocomial infections and is considered a great public health problem worldwide [1]. Although pharmacological research and development have produced a number of new antibiotics in the last three decades, bacterial resistance to these drugs has been steadily increasing [2]. Furthermore, the efficacy of antimicrobial agents currently used to treat multidrug-resistant infections is progressively declining [3]. According to Alekshun and Levy [4], the development of antibiotic-resistant bacterial strains can occur via spontaneous or induced mutagenesis or via acquisition of resistance genes from other microbial strains by horizontal gene transfer through transduction, conjugation, or transformation, resulting in selective bacterial survival and expansion in the presence of corresponding agents [4].

*S. aureus* is known to cause skin and soft-tissue infections as well as pneumonia, osteomyelitis, and abscesses [5]. Widespread use of antibiotics for the treatment of these conditions has led to the emergence and spread of drug-resistant* S. aureus* strains such as MRSA [6], which has become an important human pathogen worldwide [7]. Resistance to methicillin and other *β*-lactam antibiotics is acquired by the transfer of the* mec* A gene situated on a mobile genomic element, the staphylococcal chromosome cassette mec (SCCmec) [8]. An outbreak of MRSA infection caused by a novel phage-type of* S. aureus* occurred in three hospitals in the United States over a 21-month period [9]. Glycopeptide-type drugs such as vancomycin have proven to be effective antimicrobial agents against most MRSA strains; however, vancomycin resistance of an* S. aureus* clinical isolate was reported in Japan shortly in 1997 [10].

The mechanisms underlying antibiotic activity against various bacterial infections including those caused by* S. aureus* include interference with bacterial protein and nucleic acid synthesis, inhibition of metabolic pathways, and disruption of bacterial membrane structure and cell wall biosynthesis [10, 11]. All *β*-lactam antibiotics such as a penicillin derivative, methicillin, interfere with cell wall assembly by binding to penicillin binding-proteins (PBPs) involved in the final stages of cross-linked peptidoglycan (PGN) biosynthesis, and the discovery of PBP2a with low affinity for *β*-lactams has elucidated the mechanism underlying MRSA resistance to methicillin [11, 12]. Thus, it is very important to develop novel antibacterial agents and/or alternative therapeutic approaches to treat drug-resistant staphylococcal infections.

Several plants have demonstrated therapeutic activity against infectious diseases, but plant extracts are rarely used as systemic antimicrobial agents because of their low activity, especially in case of drug-resistant bacteria [6]. Shikonin (SKN), alkannin, and their derivatives are highly liposoluble naphthoquinone pigments isolated from the root of the plants belonging to the Boraginaceae family, including* Lithospermum erythrorhizon* Sieb. et Zucc.,* Alkanna tinctoria*,* Arnebia euchroma* (Royle) Johnst, and* Arnebia guttata* Bunge [13].* L*.* erythrorhizon* is a well-known herbal crop in Republic of Korea used in traditional oriental medicine to treat burns, ulcers, hemorrhoids, infected crusts, bedsores, external wounds, and oozing dermatitis [14, 15]. In addition,* L*.* erythrorhizon* has been reported to exhibit wound healing, anti-inflammatory, antithrombotic, and antitumor effects, and antimicrobial activity [16, 17]. Shen et al. [18] have shown that* A. euchroma*-derived SKN, which possesses antitumor activity, is also a potential candidate antimicrobial agent against MRSA and vancomycin-resistant enterococci. However, the mechanism underlying the bactericidal effects of SKN against MRSA remains unknown.

This study investigated the mechanisms of SKN antibacterial effects on the development of novel antibiotics to treat antibiotic-resistant infections. The antimicrobial activity of SKN was assessed by determining the MIC, using the broth microdilution method; then, SKN effects were further investigated in combination with membrane-permeabilizing agents, inhibitors of ATP-binding cassette (ABC) transporters, and* S. aureus*-derived PGN.

Our findings provide insights into the mechanism underlying antibacterial effects of SKN against MRSA by showing that SKN antimicrobial activity is associated with the affinity to cell wall PGN and the functional integrity of the bacterial membrane and may further the use of natural products as an alternative therapy for drug-resistant infections.

## 2. Materials and Methods

### 2.1. Plant Material and Chemicals

The roots of* L*.* erythrorhizon* were obtained from the Department of Herbal Crop Research, National Institute of Horticultural and Herbal Science, Eumsung, Chungbuk, Republic of Korea, and their identity was confirmed by Professor Nam-In Baek (Graduate School of Oriental Medicine Biotechnology, Kyung Hee University, Yongin, Republic of Korea). A voucher specimen (OMRL-090629) was deposited at the Laboratory of Oriental Medicine Research, Kyung Hee University, Yongin, Republic of Korea.

*Tris*(hydroxymethyl)aminomethane (Tris), ABC transporter-inhibiting agents sodium azide (NaN_3_) and* N*,*N*′-dicyclohexylcarbodiimide (DCCD), and ampicillin, oxacillin, and PGN were obtained from Sigma-Aldrich (St. Louis, MO, USA). Triton X-100 was purchased from Fluka (Buchs, Switzerland), and silica gel 60 (230–400 mesh) and LiChroprep RP-18 (40–63 *μ*m) were obtained from Merck (Darmstadt, Germany).

### 2.2. SKN Isolation and Analysis

Air-dried heart roots of* L. erythrorhizon* (1 kg) were powdered and extracted thrice with 2 L of aqueous 80% MeOH at room temperature for 24 h. After concentration* in vacuo*, the MeOH extract (350 g) was suspended in H_2_O (500 mL) and partitioned with pure* n*-hexane (3 × 500 mL) followed by concentration to obtain the hexane-soluble fraction (LEH, 31 g). The LEH fraction was subjected to silica gel chromatography using a 5 × 20 cm column and a gradient of* n*-hexane-EtOAc (v/v, 10 : 1 → 5 : 1 → 3 : 1 → 1 : 1, 800 mL each) to yield 10 fractions (LEH1 to LEH10). LEH5 (3.5 g) was further fractionated using a silica gel column (4 × 15 cm) and CHCl_3_-MeOH solution (v/v, 10 : 1, 53 L) to yield eight subfractions (LEH5-1 to LEH5-8). LEH5-2 (210 mg) was then separated by thin-layer chromatography using RP-18 F254s and MeOH-H_2_O solution (v/v, 1 : 1.5, 1.5 L;) to yield 71 mg SKN (*Rf*: 0.50). NMR spectrometry was performed using a Varian Unity Inova AS 400 FT-NMR instrument (Palo Alto, CA, USA), and EI mass spectrometry was performed using a JEOL JMS-700 mass spectrometer (Tokyo, Japan).

### 2.3. Bacterial Strains and Growth Conditions

Among the eight strains of* S. aureus* used in this study, six clinical MRSA isolates were obtained from six different patients at Wonkwang University Hospital (Iksan, Republic of Korea). Two other strains were* S. aureus* ATCC 33591 (MRSA) and* S. aureus* ATCC 25923 (MSSA). The ATCC 33591 and ATCC 25923 strains were purchased from the American Type Culture Collection (Manassas, VA, USA), and the remaining six MRSA strains were clinical isolates from six different patients treated at the Wonkwang University Hospital (KWMrI strains). Bacteria were stored in 10% DMSO at −80°C; for the experiments, they were suspended in Mueller-Hinton (MH) broth (Difco Laboratories, Baltimore, MD, USA) and incubated at 37°C for 48 h. The strains were maintained on MH agar plates, and antibacterial assays were performed using MH broth. Bacterial growth was monitored by measuring culture optical density (OD) at 600 nm [19, 20].

### 2.4. The Minimum Inhibitory Concentration Assay

The minimum inhibitory concentration (MIC) was determined by the broth microdilution method as described in the Clinical and Laboratory Standards Institute (CLSI) 2006 guidelines [21] using microplates and microtubes. Serial twofold dilutions (v/v) of DMSO-dissolved SKN were prepared in MH broth.* S. aureus* inoculum was adjusted to the 0.5 McFarland standard (approximately 1.5 × 10^8^ colony-forming units [CFU]/mL) in MH broth. The final inoculum was adjusted to 1.5 × 10^6^ CFU/well. The MIC was defined as the lowest SKN concentration required to visibly inhibit microbial growth after incubation at 37°C for 24 h. For further confirmation, 20 *μ*L (1 mg/mL) of 3-(4,5-dimethylthiazol-2-yl)-2,5-diphenyltetrazolium bromide (MTT) was added to* S. aureus* suspension in selected wells after 24 h incubation with SKN, for additional 20 min at 37°C; the wells developing clear yellowish color indicated the inhibition of microbial growth, and those of dark blue color indicated the absence of growth inhibition. Bacteria treated with ampicillin and oxacillin were used as negative controls.

### 2.5. The Time-Kill Test

The time-kill assay was performed as previously described [22] in 96-well microplates. Bacterial cultures were diluted with fresh MH broth to ~1 × 10^6^ CFU/mL and incubated at 37°C. Culture aliquots (100 *μ*L) were taken at 0, 4, 8, 12, and 24 h, serially 10-fold diluted in MH broth, and spread on drug-free MH agar plates. After 24 h incubation at 37°C,* S. aureus* colonies (up to 300) were counted on each plate. The lower limit of sensitivity for colony counts was 100 CFU/mL. The time-kill assay was performed at least thrice; the data are represented as mean data ± standard deviation (S.D.).

### 2.6. SKN Synergy with Membrane-Binding Agents and ATPase Inhibitors

To determine whether SKN antibacterial activity was associated with membrane function, SKN was used in combination with membrane-permeabilizing chemicals Tris and Triton X-100 or ATPase-inhibiting agents DCCD and NaN_3_, which can decrease ATP levels by disrupting electrochemical proton gradients in bacteria [20]. The membrane-permeabilizing agents and ATPase inhibitors were added at concentrations that did not inhibit bacterial growth: 0.01% for Tris, Triton X-100, and NaN_3_; and 0.005% for DCCD; similarly, SKN was used at 0.25 MIC, which did not significantly affect MRSA viability. The cultures were incubated at 37°C for 24 h, and their growth was evaluated by measuring OD600 using a microplate reader.

### 2.7. SKN Binding to Peptidoglycan

To determine whether SKN directly bound PGN in* S. aureus* cell wall, SKN (7.8 *μ*g/mL, 0.5 MIC) was added together with PGN (1.9–125 *μ*g/mL) to MRSA cultures as previously described [23]. Lipopolysaccharide (LPS) was used as negative control; it did not demonstrate inhibitory activity against MRSA. The cultures were treated at 37°C for 24 h, and their growth was evaluated by measuring OD600 using a microplate reader.

### 2.8. Transmission Electron Microscopy (TEM)

MRSA exponential-phase cultures were obtained by diluting overnight cultures with MH broth to the 0.5 McFarland standard (approximately 1.5 × 10^8^ colony-forming units [CFU]/mL) in MH broth. The inoculum was then diluted tenfold (v/v) in MH broth. When MRSA cultures reached the midlogarithmic phase (approximately 1.5 × 10^6^ colony-forming units [CFU]/mL), they were treated with 7.8 *μ*g/mL and 15.6 *μ*g/mL of SKN for 10 h. Then, 2 mL of the cultures was centrifuged at 10,000 ×g for 10 min, and the pellets were treated with Karnovsky's fixative and examined under an energy-filtering transmission electron microscope (LIBRA 120; Carl Zeiss, Oberkochen, Germany) at an accelerating voltage of 100 kV. Images were obtained using a 4 k × 4 k slow-scan charge-coupled device camera (Ultrascan 4000 SP; Gatan, Pleasanton, CA, USA) attached to the microscope [23, 24].

### 2.9. Statistical Analysis

All experiments were performed at least three times, and the data are presented as the mean ± S.D. The data were analyzed using one-way analysis of variance (ANOVA), and the differences among groups were evaluated using Dunnett's multiple comparisons test. *P* < 0.05 was considered statistically significant [25].

## 3. Results

### 3.1. SKN Isolation and Characterization

**Figure 1:**
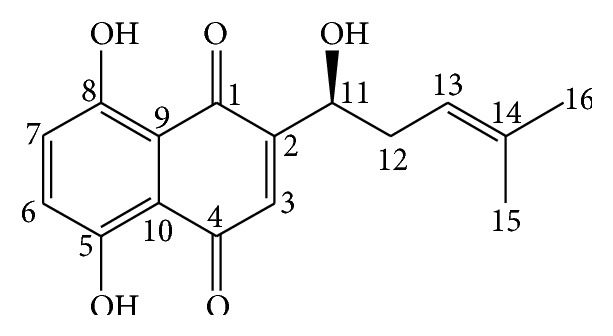
Chemical structure of shikonin.

SKN was isolated from the MeOH extract of* L. erythrorhizon* roots and identified by spectral analysis (Table 1). SKN represented red crystals and exhibited a UV absorption maximum at 292 nm. The molecular mass was determined as 288 [M]^+^ based on the EI/MS data. The ^1^H-NMR (400 MHz, CDCl_3_) spectrum showed four olefin methine signals due to double bonds at *δ*_H_ 7.16 (H-6 and H-7), 7.12 (H-3), and 5.18 (H-13). Signals due to an oxygenated methine proton at *δ*_H_ 4.88 (1H, dd, *J* = 7.6, 4.2 Hz, H-11), methylene protons at *δ*_H_ 2.62 (H-12a) and 2.34 (H-12b), and methyl protons at *δ*_H_ 1.73 (H-16) and 1.60 (H-15) were observed. The ^13^C-NMR spectrum and DEPT showed 16 carbon signals. In the low magnetic field, two carbonyl group carbons at *δ*_C_ 180.0 (C-1) and 180.3 (C-4), two olefin carbons at *δ*_C_ 132.0 (C-6) and 132.1 (C-7), and four olefin quaternary carbons at *δ*_C_ 165.3 (C-5), 164.7 (C-8), 112.3 (C-9), and 111.2 (C-10) of a benzene ring were detected. Accordingly, SKN was assumed to be a naphthoquinone. In addition, one oxygenated methine at *δ*_C_ 68.0 (C-11), one methylene at *δ*_C_ 35.2 (C-12), and two methyl signals at *δ*_C_ 18.0 (C-15) and 25.7 (C-16) were observed (Figure 1). The chemical structure of SKN obtained based on 1D and 2D-NMR spectroscopic data and MS data was confirmed by comparison with previous findings [26].

### 3.2. SKN Activity against MRSA

Antimicrobial susceptibility tests of SKN against eight strains of* S. aureus* were performed using the standard broth microdilution method. SKN MIC against MSSA strain was 7.8 *μ*g/mL, and against seven MRSA strains it ranged from 7.8 to 31.2 *μ*g/mL. Ampicillin and oxacillin, used as negative controls, exhibited MICs of 0.9 *μ*g/mL and 1.9 *μ*g/mL against the MSSA strain, respectively, whereas their activity against the seven MRSA strains was much lower: 31.2–250 *μ*g/mL and >250 *μ*g/mL, respectively (Table 2).

**Figure 2:**
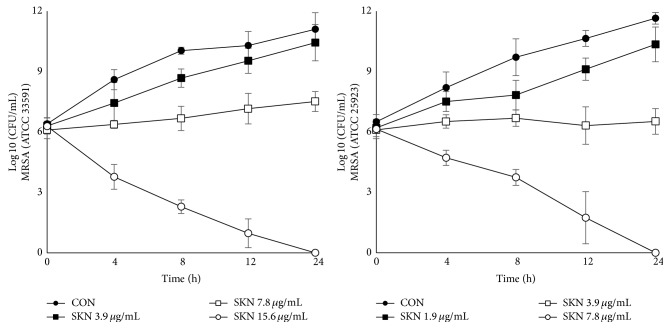
Time-kill curves of MRSA (ATCC 33591) and MSSA (ATCC 25923) strains treated with shikonin (SKN).

The time-kill assay was used to determine the bactericidal or bacteriostatic activity of SKN for the MSSA and MRSA (ATCC 33591) strains. SKN showed progressive concentration-dependent inhibitory activity against both strains. For the MRSA strain, SKN concentrations of 3.9, 7.8, and 15.6 *μ*g/mL (corresponding to 0.25 MIC, 0.5 MIC, and MIC, resp.) were bacteriostatic with an initial decrease in viable cell counts (Figure 2). SKN at the MIC (15.6 *μ*g/mL) and 0.5 MIC (7.8 *μ*g/mL) inhibited the growth of MRSA and MSSA, respectively, over a 24 h period.

### 3.3. SKN Antibacterial Activity Is Enhanced by Membrane-Binding Agents and ATPase Inhibitors

**Figure 3:**
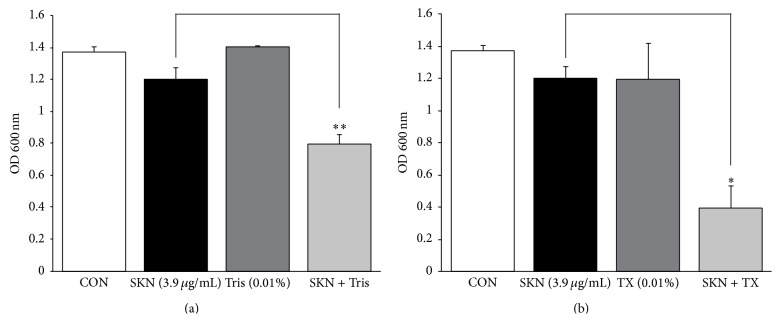
The effect of membrane-permeabilizing agents on the susceptibility of MRSA (ATCC 33591) to shikonin (SKN). Bacterial viability was determined by the absorbance at 600 nm after incubation for 24 h with SKN (3.9 *μ*g/mL, 0.25 MIC value), 0.01% Tris (a), and 0.01% Triton X-100 (TX, (b)), or their combinations SKN + Tris and SKN + TX, respectively. The data are presented as the mean ± S.D. of three independent experiments. ^*∗*^ *P* < 0.05 and ^*∗∗*^ *P* < 0.005 compared to SKN alone. CON, untreated control MRSA.

**Figure 4:**
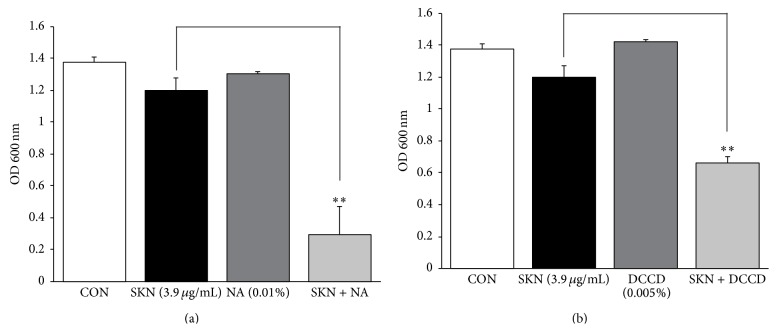
Combined effect of ATPase inhibitors on the susceptibility of MRSA (ATCC 33591) to shikonin (SKN). Bacterial viability was determined by the absorbance at 600 nm after incubation for 24 h with SKN (7.8 *μ*g/mL, 0.5 MIC value), 0.01% NaN_3_ (NA, (a)), and 0.005%* N*,*N*′-dicyclohexylcarbodiimide (DCCD, (b)), or their combinations SKN + NA and SKN + DCCD, respectively. The data are presented as the mean ± S.D. of three independent experiments. ^*∗∗*^ *P* < 0.005 compared to SKN alone. CON, untreated control MRSA.

To determine the effects of membrane permeability on SKN antistaphylococcal activity, the MRSA (ATCC 33591) strain was treated with the combination of SKN (0.25 MIC) and membrane-permeabilizing agents Tris (0.01%) and Triton X-100 (0.01%). In the used concentrations, Tris, Triton X-100, or SKN alone did not significantly affect MRSA growth; however, SKN + Tris and SKN + Triton X-100 decreased MRSA growth by 34% and 67%, respectively, compared to SKN alone (Figure 3). Furthermore, MRSA viability decreased dramatically after the treatment with SKN in combination with ATPase inhibitors NaN_3_ (0.01%) and DCCD (0.005%): to 75% and 45%, respectively, compared to the cultures treated with SKN (0.25 MIC) alone (Figure 4).

### 3.4. *S. aureus* PGN Inhibits SKN Activity against MRSA

**Figure 5:**
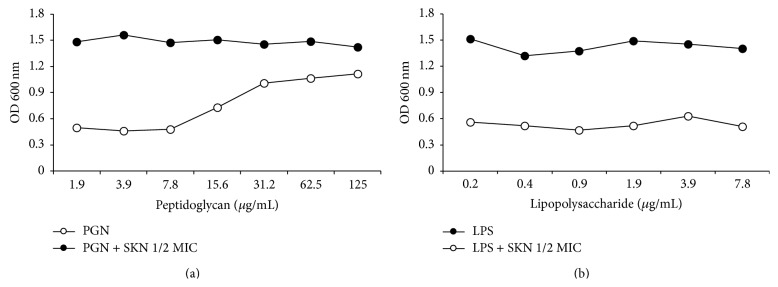
*S. aureus* cell wall peptidoglycan (PGN) inhibits the antimicrobial activity of shikonin (SKN) against the MRSA (ATCC 33591) strain. MRSA cultures were treated with SKN (7.8 *μ*g/mL, 0.5 MIC) in the presence of indicated concentrations of PGN (a) or lipopolysaccharide (LPS, (b)) at 37°C for 24 h; cell viability was determined by the absorbance at 600 nm.

Next, we examined whether SKN might directly bind to the cell wall and interfere with its integrity, by treating MRSA with SKN (0.5 MIC) in the presence of* S. aureus* PGN (0–125 *μ*g/mL); LPS was used as negative control. PGN at the concentration of 125 *μ*g/mL inhibited the antibacterial activity of SKN, whereas LPS did not show any effect (Figure 5), suggesting that SKN may directly bind* S. aureus* PGN.

### 3.5. SKN Treatment Alters MRSA Morphology

**Figure 6:**
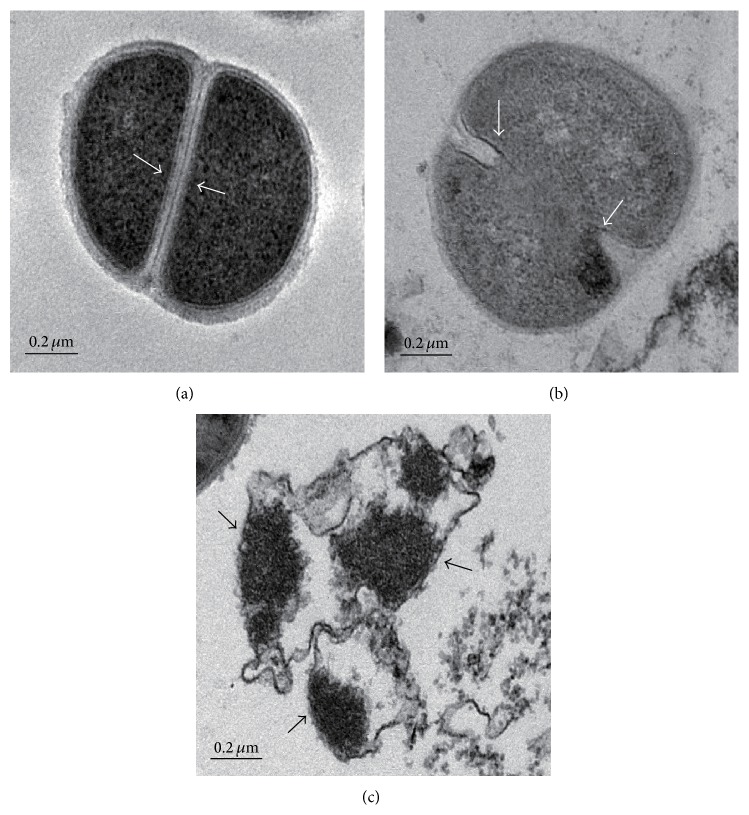
Transmission electron microscopy images of SKN-treated MRSA (ATCC 33591) cells. (a) Untreated control MRSA cells; white arrows indicate intact cell membrane. (b) MRSA cells treated with SKN (7.8 *μ*g/mL, 0.5 MIC) for 10 h; white arrows indicate significant membrane damage. (c) MRSA cells treated with SKN (15.6 *μ*g/mL, MIC); black arrows indicate disrupted bacterial membrane and dispersed intracellular contents.

To determine whether SKN affected MRSA morphology, SKN-treated bacterial cells were examined by TEM. The results indicated that SKN caused ultrastructural changes in the cells of MRSA (ATCC 33591) strain. Thus, the untreated bacterial cells had normal morphology with distinct septa (Figure 6(a)). However, 24 h exposure to SKN at 0.5 MIC caused disruption of the cytoplasmic membrane (Figure 6(b)), whereas exposure to SKN at the MIC caused complete membrane disintegration, cell lysis, and release of cytoplasmic contents (Figure 6(c)).

## 4. Discussion

The increasing emergence of multidrug-resistant bacteria is a global problem. Few new drugs are available against MRSA strains, which are resistant to most antibiotics [27]. Therefore, the search for new and effective antimicrobial agents and/or novel therapeutic approaches for the treatment of infectious diseases caused by drug-resistant bacteria including MRSA is extremely important. Recently, there has been considerable interest among medical professionals in the application of herbal medicines as alternative methods to control pathogenic microorganisms [28].* L. erythrorhizon* extract has been used in oriental traditional medicine because of its antibacterial, anti-inflammatory, immunostimulating, antitumor, and wound-healing activities [29]; however, the mechanism underlying SKN antibacterial effects has not been investigated.

In this study, we indicated that SKN, at concentrations much lower than the MIC, significantly reduced MRSA growth if used together with membrane-permeabilizing compounds or ATPase inhibitors.

Tris and Triton X-100 have been shown to augment cell membrane permeability, reduce methicillin resistance, and stimulate cell autolysis [30]; they have also potentiated the anti-MRSA activity of a flavonoid compound isolated from the roots of* Desmodium caudatum* [24]. The membrane-permeabilizing activity of Tris and Triton X-100 increased MRSA susceptibility to SKN, while 0.01% Tris or 0.01% Triton X-100 alone had no effect on cell viability.

NaN_3_ and DCCD are inhibitors of ATP synthase in bacterial cells [25, 31]. DCCD disturbs the H^+^-translocating sector (F_0_) of the F_0_F_1_-ATP synthase in coupling membranes [32]. Most bacteria produce ABC transporters, which comprise an essential uptake system for amino acids in bacteria and are shown to be involved in multidrug resistance including that of MRSA [23, 31]. NaN_3_ is known to inhibit ATP synthesis in mitochondria and ATP-dependent transport systems such as endocytosis by disrupting electrochemical proton gradients in bacterial cells [20, 24, 33].

These results show that SKN anti-MRSA effect was potentiated by both DCCD and NaN_3_, suggesting that SKN antimicrobial activity is associated with cytoplasmic membrane permeability and inhibition of ATPase function. Our findings are consistent with those of previous studies showing that detergents and ATPase inhibitors significantly increased MRSA susceptibility to natural antimicrobial agents such as sophoraflavanone B from* D. caudatum*, tectorigenin from* Belamcanda chinensis*, and epigallocatechin gallate from* Camellia sinensis* [19, 23, 24].

PGN and lipoteichoic acid are main components of the cell wall of gram-positive bacteria including MRSA [34]. The cell wall of gram-positive bacteria, consisting of up to 30 PGN layers, plays an essential role not only in osmotic protection but also in cell division [23]. The cell wall of gram-positive bacteria contains a very thick layer of cross-linked PGN, whereas in gram-negative bacteria the PGN layer is thin and overlaid by the outer membrane composed mainly of LPS [35, 36]. As shown in Figure 5, SKN alone (7.8 *μ*g/mL) significantly inhibited MRSA growth, but MRSA-derived PGN (125 *μ*g/mL) blocked the antibacterial activity of SKN, suggesting that PGN, by directly binding to SKN, prevented it from damaging the MRSA cell wall.

TEM can provide useful insights into the mechanism underlying the activity of antibacterial agents by directly demonstrating the morphological changes induced in bacterial cells [23]. Antimicrobial compounds can damage the bacterial cell wall and cytoplasmic membrane, causing cell lysis and leakage of cytoplasmic content [36, 37]. TEM images of SKN-treated MRSA demonstrated cytoplasmic membrane disruption and cell lysis, followed by the leakage of intracellular components, thereby confirming that SKN has anti-MRSA activity.

This study, showing the antibacterial effects of SKN in combination with membrane-permeabilizing agents or ATPase inhibitors, suggests that SKN isolated from* L. erythrorhizon* acts by binding to PGN of MRSA and may be a promising candidate antibacterial compound for the treatment of multidrug-resistant* S. aureus* infections. However, more studies are required to confirm the antibacterial activity of SKN against MRSA in vivo and to assess its potential for clinical application in MRSA-infected patients. Future studies will also include an investigation of SKN toxicity and bioavailability and combination tests with existing antibiotics.

## Figures and Tables

**Table 1:** ^1^H-NMR (400 MHz) and ^13^C-NMR (100 MHz) data of SKN (in CDCl_3_, *δ* in ppm, *J* in Hz)^a^.

Number	*δ*_H_	*δ*_C_
1		180.0
2		151.2
3	7.12 (1H, s)	131.6
4		180.3
5		165.3
6	7.16 (1H, s)	132.0
7	7.16 (1H, s)	132.1
8		164.7
9		112.3
10		111.2
11	4.88 (1H, dd, *J* = 7.6, 4.2 Hz)	68.0
12	2.62 (1H, m, H-12a)	35.2
	2.34 (1H, m, H-12b)	
13	5.18 (1H, m)	118.1
14		137.3
15	1.60 (3H, s)	18.0
16	1.73 (3H, s)	25.7

^a^Assignments were confirmed by 1D (DEPT) and 2D-NMR (HSQC, HMBC).

**Table 2:** Antibacterial activity of shikonin (SKN), ampicillin, and oxacillin against eight *S*. *aureus* strains.

Bacterial strains	Class	*mec A* gene	*β*-lactamase activity	MIC (*μ*g/mL)		
				SKN	Ampicillin	Oxacillin
ATCC 33591	MRSA	+	+	15.6	125	>250
ATCC 25923	MSSA	−	−	7.8	0.9	1.9
Clinical isolates						
KWMrI 1039^a^	MRSA	+	+	7.8	31.2	>250
KWMrI 1040	MRSA	+	−	15.6	31.2	>250
KWMrI 1041	MRSA	+	+	15.6	62.5	>250
KWMrI 1046	MRSA	+	+	31.2	250	>250
KWMrI 1047	MRSA	+	+	7.8	125	>250
KWMrI 1048	MRSA	+	−	31.2	62.5	>250

MSSA, methicillin-susceptible *S*. *aureus*; MRSA, methicillin-resistant *S*. *aureus*; KWMrI (a), MRSA strains from the Department of Plastic Surgery, Wonkwang University Hospital.
